# Gleiche Chancen beim Zugang zur medizinischen Rehabilitation? – Ein Vergleich zweier qualitativer Studien

**DOI:** 10.1007/s00103-026-04268-w

**Published:** 2026-07-10

**Authors:** Maria Mader, Lia Seibel, Stefanie Neudecker, Jana Stucke, Anna Brinkmann, Oliver Razum

**Affiliations:** 1 Fakultät für Gesundheitswissenschaften, AG3 Epidemiologie & International Public Health, Universität Bielefeld, Universitätsstr. 25, 33615 Bielefeld, Deutschland; 2 Medizinische Fakultät OWL, AG Medizinische Versorgung von Menschen mit Behinderung und chronischen Erkrankungen, Universität Bielefeld, Bielefeld, Deutschland

**Keywords:** Chancengleichheit, Zugang zu medizinischer Rehabilitation, Menschen mit vorbestehenden Behinderungen, Herausforderungen, Qualitative Studien, Equal opportunities, Access to medical rehabilitation, People with pre-existing disabilities, Challenges, Qualitative studies

## Abstract

**Einleitung:**

Der Zugang zur medizinischen Rehabilitation ist in Deutschland weitgehend einheitlich geregelt, wird aber durch verschiedene Rahmenbedingungen beeinflusst. Die vorliegende Arbeit untersucht, inwieweit bei Menschen mit vorbestehenden Behinderungen Unterschiede im Zugang zur medizinischen Rehabilitation im Vergleich zu allen Menschen mit Rehabilitationsbedarf bestehen.

**Methoden:**

Zwei qualitative Studien wurden ausgewertet: MeReMBe (Medizinische Rehabilitation für Menschen mit einer vorbestehenden Behinderung) (02/2024–01/2026) und FöBeR (Förderliche Faktoren bei der Beantragung einer medizinischen Rehabilitation)  (04/2021–08/2022). In beiden Studien erfolgte ein Maximum-Variation-Sampling. Insgesamt wurden 19 Expert*inneninterviews und 8 Fokusgruppen mit Prozessbeteiligten durchgeführt. Die Daten wurden transkribiert, pseudonymisiert und mit der inhaltlich strukturierenden Inhaltsanalyse ausgewertet.

**Ergebnisse:**

Anträge zur Rehabilitation werden häufig durch An- und Zugehörige sowie Hausärzt*innen initiiert, die damit eine zentrale Rolle im Zugangsprozess übernehmen. Besonders für Menschen mit komplexen Beeinträchtigungen oder erhöhtem Pflegebedarf ist dies relevant. Aufgrund von beispielsweise kognitiven und Kommunikationsbeeinträchtigungen ist ihnen eine gewohnte kommunikative Entscheidungsfindung oft nicht möglich. Zusätzlich erschwert die indikationsorientierte Struktur des Rehabilitationssystems den Zugang, weil komplexe oder multiple Beeinträchtigungen häufig nicht eindeutig einer einzelnen Indikation zugeordnet werden können.

**Diskussion:**

Zur Verbesserung des Zugangs braucht es niedrigschwellige Informationen und Beratung für potenzielle Rehabilitand*innen und Prozessbeteiligte. Diversitätsbezogene Faktoren wirken im Zugangsprozess zusammen und können sich verstärken (z. B. Behinderung und Migrationshintergrund). Komplexe bzw. besondere Bedarfe werden auch hier unzureichend berücksichtigt und erschweren trotz gleicher Chancen den Zugang zur Versorgung.

## Abstract (en)

**Background:**

Access to medical rehabilitation in Germany is largely standardized, yet influenced by various contextual conditions. This study examines the extent to which people with pre-existing disabilities face differences in access to medical rehabilitation compared with all people in need of rehabilitation.

**Methods:**

Two qualitative studies were analyzed: MeReMBe (Medizinische Rehabilitation für Menschen mit einer vorbestehenden Behinderung) (2/2024–1/2026) and FöBeR(Förderliche Faktoren bei der Beantragung einer medizinischen Rehabilitation) (04/2021–08/2022). Both studies used maximum variation sampling. A total of 19 expert interviews and 8 focus groups with stakeholders involved in the rehabilitation process were conducted. The data were transcribed, pseudonymized, and analyzed using structuring content analysis.

**Results:**

Applications to rehabilitation are often initiated by relatives and other close persons as well as by general practitioners, who play a key role in the access process. Due to cognitive and communication impairments, for example, the usual communicative decision-making process is often not possible. In addition, the indication-oriented structure of the rehabilitation system impedes access because complex or multiple impairments often cannot be clearly assigned to a single indication category.

**Discussion:**

To support access to medical rehabilitation, potential rehabilitation patients and those involved in the process (e.g., relatives and healthcare professionals) require more low-threshold information and counseling options to fully benefit from the potential offered by medical rehabilitation. Diversity-related factors can interact in the access process and reinforce each other (e.g., disability and migration background). Complex or specific needs are insufficiently considered here, making it difficult to provide access to care.

## Einleitung

Die medizinische Rehabilitation (Reha) ist angesichts des demografischen und epidemiologischen Wandels in Deutschland eine relevante Gesundheitsstrategie, um Multimorbidität, Einschränkungen der Funktionsfähigkeit und Behinderung entgegenzuwirken [1–3]. Das Ziel der medizinischen Reha ist, die Teilhabe in der Gesellschaft und am Arbeitsleben zu fördern, gesundheitliche Beeinträchtigungen abzumildern sowie die Lebensqualität zu verbessern [4, 5]. Der Zugangsprozess zur medizinischen Reha ist im Sozialgesetzbuch IX (SGB IX) verankert. Er umfasst die Feststellung des Reha-Bedarfs und die anschließende Antragstellung. Der Antrag wird dann bei einem zuständigen Leistungsträger, z. B. Deutsche Rentenversicherung (DRV) oder Gesetzliche Krankenversicherung (GKV), eingereicht. Die Leistungsträger ermitteln den Reha-Bedarf anhand der Unterlagen und erlassen einen Leistungsbescheid. Die sozialmedizinischen Kriterien zur Erkennung eines Reha-Bedarfs umfassen Reha-Bedürftigkeit, Reha-Fähigkeit und eine positive Reha-Erfolgsprognose [1, 4]. Je nach Leistungsträger unterscheidet sich das Antragsverfahren: In der GKV erfolgt es häufig über eine ärztliche Verordnung (z. B. aus der ambulanten Versorgung) oder als im Krankenhaus beantragte Anschlussrehabilitation, während bei der DRV u. a. eine Selbstauskunft der Versicherten sowie ein ärztlicher Befundbericht erforderlich sind [1, 4, 5]. Der Zugang zur rehabilitativen Versorgung wird durch viele Faktoren und Entscheidungen Prozessbeteiligter beeinflusst. Neben einer Erkrankung der potenziellen Rehabilitand*innen (z. B. chronische Herz-Kreislauf-Erkrankung) und deren Folgen für die Teilhabe am Erwerbsleben und Gesellschaft spielen auch verschiedene Voraussetzungen, Kompetenzen und Diversitätsmerkmale/soziale Determinanten (z. B. Lebenslagen, sozioökonomischer Status und vorbestehende Behinderungen) eine Rolle. Darüber hinaus beeinflusst auch das administrative und institutionelle Verfahren den Zugangsprozess zur rehabilitativen Versorgung [1, 6].

In Deutschland leben etwa 7,9 Mio. Menschen mit einer amtlich anerkannten Behinderung. Davon sind ca. 4,5 Mio. Menschen von einer körperlichen, 1,8 Mio. von einer zerebralen, geistigen oder seelischen und ca. 1,5 Mio. von einer nicht näher bezeichneten Behinderung betroffen. Zu den Ursachen einer schwerwiegenden Behinderung zählen bspw. angeborene Behinderungen und Arbeitsunfälle [7].

Die Versorgung von Menschen mit Behinderungen stellt eine zentrale Herausforderung für das Gesundheitssystem dar [8–11]. Die Behindertenrechtskonvention der Vereinten Nationen (UN-BRK) bildet eine menschenrechtliche Grundlage dafür. Sie verfolgt das Ziel der Chancengleichheit und des Rechts auf Gesundheit für Menschen mit Behinderungen (vgl. Art. 25). Die Vertragsstaaten erkennen das Recht von Menschen mit Behinderungen auf das erreichbare Höchstmaß an Gesundheit ohne Diskriminierung an. Sie verpflichten sich, geeignete Maßnahmen zu ergreifen, um einen gleichberechtigten Zugang zu Gesundheitsleistungen und Rehabilitation sicherzustellen. In Deutschland sind diese Ziele seit 2009 auch gesetzlich verankert [8–10]. Dennoch bestehen weiterhin Ungleichheiten und Barrieren in der Gesundheitsversorgung: Menschen mit Behinderungen weisen im Vergleich zur Allgemeinbevölkerung einen schlechteren Gesundheitszustand und einen schlechteren Zugang zu Gesundheitsdiensten auf [8, 9, 12]. Daneben haben sie ein höheres Risiko für chronische, schwere oder psychische Erkrankungen sowie Multimorbidität, woraus sich ein potenziell höherer Bedarf an medizinischer Reha ergibt [9, 13–17]. Gleichzeitig gibt es Hinweise darauf, dass Menschen mit Behinderungen vor ähnlichen Herausforderungen stehen, wie Menschen, deren andere Diversitätsmerkmale in der Gesundheitsversorgung wenig Berücksichtigung finden [18, 19].

Ziel dieses Beitrags ist es daher, am Beispiel von Menschen mit vorbestehenden Behinderungen (MmvB) zu untersuchen, inwieweit Unterschiede und ähnliche Herausforderungen im Zugang zur medizinischen Reha im Vergleich zu allen Menschen mit Reha-Bedarf (MRb) bestehen.

## Methoden

In dieser Arbeit erfolgt ein Vergleich zweier explorativer, qualitativer Studien über eine qualitative Synthese [20]. Beide Studien wurden an der Fakultät für Gesundheitswissenschaften, Universität Bielefeld durchgeführt.

Bei der einen Studie handelt es sich um das Projekt *MeReMBe* (Medizinische Rehabilitation für Menschen mit einer vorbestehenden Behinderung). Es fand im Zeitraum 01.02.2024–31.01.2026 statt. Ziel war es, zu untersuchen, wie der Prozess der medizinischen Reha sich für MmvB (mit Beginn der Antragstellung bis hin zur Inanspruchnahme) gestaltet. MmvB werden definiert als Personen, die mit einer Behinderung leben und aufgrund einer Sekundärerkrankung, einer Komorbidität oder eines Unfalls einen Rehabilitationsbedarf haben, d. h. wegen eines zusätzlich auftretenden Gesundheitsproblems. Teile der in dieser Studie erhobenen Ergebnisse sind zudem in eine Dissertationsarbeit eingeflossen und wurden dort ausführlicher dargestellt [21]. Zum Vergleich dient die Studie *FöBeR* (Förderliche Faktoren bei der Beantragung einer medizinischen Rehabilitation). Darin wurde im Zeitraum 01.04.2021–31.08.2022 untersucht, wie förderliche Faktoren den Zugang zur medizinischen Rehabilitation bei MRb beeinflussen. Insgesamt wurden 8 Fokusgruppen sowie 19 Interviews mit Expert*innen durchgeführt.

Eine Kurzdarstellung der Methoden beider Studien ist in Tab. 1 aufgeführt.

**Tab. 1:** Methodenvergleich der beiden ausgewerteten Studien

	FöBeR	MeReMBe
Zielgruppe	Alle Menschen mit Rehabilitationsbedarf	Menschen mit vorbestehender Behinderung (MmvB) mit Rehabilitationsbedarf
Rekrutierung	Per E‑Mail, telefonisch und via Gatekeeper	
Methode der Erhebung	Expert*inneninterviews und Fokusgruppen	Expert*inneninterviews, Gruppendiskussionen und Interviews mit MmvB
Leitfadenentwicklung	SPSS-Methode	
Sampling	Maximum Variation	
Auswertung	Strukturierende Inhaltsanalyse	

### Rekrutierung und Setting beider Studien

Beide Studien verfolgten ein Maximum-Variation-Sampling, um möglichst unterschiedliche Sichtweisen von Expert*innen im Zugang zur medizinischen Rehabilitation abzubilden [20]. Ziel war es, Gesundheitsprofessionelle und weitere Prozessbeteiligte aus verschiedenen Versorgungssektoren und andere informationsreiche Expert*innen zu erreichen. Die Rekrutierung erfolgte über E‑Mail, telefonisch oder via Gatekeeper. Eine Sättigung trat in beiden Studien ein, als weitere Erhebungen zu keinen neuen Informationen bzw. zu einer Redundanz von Informationen führten [20, 21].

#### Besondere Aspekte der FöBeR-Studie.

In der FöBeR-Studie wurden qualitative Interviews und (Online‑)Fokusgruppen mit Expert*innen durchgeführt. Die Expert*innen für den Zugang zur und der Beantragung der medizinischen Reha wurden z. B. über das Netzwerk des NRW-Forschungsverbunds für Reha-Wissenschaften, Selbsthilfevertretungen auf Landesebene (u. a. NRW) und Mediziner*innen über das Netzwerk der Allgemein- und Familienmedizin der Medizinischen Fakultät Bielefeld rekrutiert. Teilnehmende Expert*innen waren z. B. Gesundheitsprofessionelle aus ambulanter, stationärer (z. B. medizinische Fachangestellte, Casemanager*innen) und rehabilitativer Versorgung sowie Forschende aus der Reha- und Versorgungsforschung (Tab. 2).

**Tab. 2:** Berufsgruppen der interviewten Teilnehmenden der FöBeR- und MeReMBe-Studie

Sektor	Berufs- und Handlungsfelder	Anzahl	
		FöBeR	MeReMBe
Ambulanter Sektor (Primärversorgung)	Medizinische Berufe	3	4
	Therapeutische Berufe	0	1
	Projekt- und Qualitätsmanagement	1	0
	Casemanagement	2	1
	Soziale Berufe	0	4
Stationärer Sektor (Primärversorgung)	Medizinische Berufe	1	0
	Casemanagement	3	0
	Soziale Berufe	0	1
Rehabilitativer Sektor	Medizinische Berufe	8	2
	Therapeutische Berufe	8	0
	Projekt- und Qualitätsmanagement	1	0
	Soziale Berufe	1	0
Sonstige	Leistungsträger	2	3
	Selbsthilfe	3	0
	Hochschulkontext	1	0
	Unbekannt	1	0

#### Besondere Aspekte der MeReMBe-Studie.

Im Rahmen der *MeReMBe*-Studie wurden u. a. leitfadengestützte Interviews mit Expert*innen zu ihren Erfahrungen mit dem Prozess der medizinischen Rehabilitation von MmvB durchgeführt. Dabei wurden Personen angefragt, die im Rehabilitationsprozess von MmvB eine Rolle einnehmen. Im Vorfeld wurden diese potenziellen Prozessbeteiligten in 4 Samplinggruppen unterteilt: (1.) informelle Unterstützende (z. B. Angehörige, Unterstützende aus der Community), (2.) formalisierte Unterstützende (z. B. Sozialarbeitende, Mitarbeitende der ergänzenden unabhängigen Teilhabeberatung, Mitarbeitende der Eingliederungshilfe, Mediziner*innen), (3.) Leistungserbringende in Rehakliniken (z. B. Therapeut*innen, Pflegekräfte, Mediziner*innen, Verwaltungsmitarbeitende) sowie (4.) Kostenträger (z. B. Mitarbeitende der DRV, GKV). Ziel war es, eine möglichst heterogene Gruppe von potenziellen Prozessbeteiligten aus unterschiedlichen Gesundheitssektoren zu rekrutieren.

### Datenerhebung beider Studien

Die Entwicklung der Interviewleitfäden erfolgte für *FöBeR* und *MeReMBe* nach der SPSS-Methode („Sammeln“, „Prüfen“, „Sortieren“ und „Subsummieren“) von Helfferich [22]. Die Leitfäden wurden jeweils in Forschungswerkstätten und von Kolleg*innen geprüft, anschließend jeweils in einem Pilot-Interview (*MeReMBe*) oder einer (Online‑)Fokusgruppe *(FöBeR*) getestet und iterativ weiterentwickelt. Tab. 3 zeigt die Themen der Leitfäden. Die Erhebungen, Verarbeitung und Auswertung der Daten erfolgte konform mit der Datenschutz-Grundverordnung (DSGVO). Einige Fokusgruppen und Interviews wurden via Zoom unter Ende-zu-Ende-Verschlüsselung durchgeführt. Alle Teilnehmenden gaben eine informierte Einwilligung.

**Tab. 3:** Leitfädenthemen beider Studien bzgl. Herausforderungen bei der Beantragung und dem Zugang zur medizinischen Rehabilitation

FöBeR	MeReMBe
Einflussfaktoren bei (k)einem Zugang und Antragstellung zur medizinischen Rehabilitation	Einflussfaktoren zur Feststellung eines Rehabilitationsbedarfs
Gründe für (k)einen Zugang und Antragstellung	Gründe für oder gegen einen Antrag zur medizinischen Rehabilitation
Charakteristika der Menschen mit Rehabilitationsbedarf	Indikationen eines Rehabilitationsbedarfs
Bedingungen, Voraussetzungen und Herausforderungen für den Zugang	Herausforderungen beim Zugang im Rehabilitationsprozess

*Anmerkung: *Trotz unterschiedlicher Benennung der Themen in den Studien zeigen diese eine hohe inhaltliche Übereinstimmung und sind daher miteinander vergleichbar

#### Besondere Aspekte der FöBeR-Studie.

Die leitfadengestützten Erhebungen wurden bei Bedarf persönlich, telefonisch, via Zoom oder vor Ort in medizinischen Einrichtungen durchgeführt und dauerten zwischen 21 min und 80 min. Die Erhebungen erfolgten zwischen 2021 und 2022. Insgesamt haben *n* = 36 Personen teilgenommen. Es wurden *n* = 5 Fokusgruppen in Präsenz, *n* = 3 Online-Fokusgruppen via Zoom und sowie *n* = 3 Interviews durchgeführt. Die Anzahl der Teilnehmenden lag bei 3–5 Expert*innen je (Online‑)Fokusgruppe. Die Aussagen der Expert*innen wurden während der (Online‑)Fokusgruppen mithilfe von Moderationskarten und -wänden diskutiert.

#### Besondere Aspekte der MeReMBe-Studie.

Die Expert*inneninterviews fanden persönlich, telefonisch, via Zoom oder vor Ort statt. Die Interviews dauerten zwischen 30 min und 117 min und wurden zwischen 2024 und 2025 durchgeführt. Insgesamt nahmen *n* = 16 Expert*innen an den Interviews teil. Dabei wurden *n* = 4 Interviews telefonisch, *n* = 6 via Zoom und *n* = 6 persönlich am Arbeitsplatz der Teilnehmenden durchgeführt.

### Datenauswertung beider Studien

In beiden qualitativen Studien wurden die Interviews auditiv aufgezeichnet, nach Dresing und Pehl [23] transkribiert und pseudonymisiert. Daraufhin erfolgte jeweils eine deduktiv-induktive, kategorienbasierte Auswertung des Textmaterials mit der inhaltlich strukturierenden qualitativen Inhaltsanalyse nach Kuckartz und Rädiker [24] in MAXQDA. Die Transkripte wurden im Rahmen verschiedener Forschungswerkstätten diskutiert und anhand der Forschungsfragen induktiv kodiert.

Für den Vergleich der beiden Studien wurden aus den Themenbereichen der Leitfäden (Tab. 3) die in Tab. 4 dargestellten Kategorien ausgewählt. Diese beziehen sich auf die Herausforderungen bei der Beantragung und dem Zugang zur medizinischen Rehabilitation. Die in Tab. 4 dargestellten Kategorien unterscheiden sich zwischen den Studien in der Formulierung, sind inhaltlich aber vergleichbar.

**Tab. 4:** Genutzte Kategorien für den Vergleich beider Studien

Für den Artikel genutzte Hauptkategorien	
MeReMBe	FöBeR
Zugangswege zur Rehabilitation	Zugangswege zur medizinischen Rehabilitation
Barrieren im Zugang	Herausforderungen im Zugang zur Reha
Ablauf der Antragstellung	Bedingungen für einen Rehabilitationsantrag
Klinikwahl	Bedingungen für einen Rehabilitationsantrag
Zuständigkeiten der Kostenträger/Prozessbeteiligten	Akteure im Antragsprozess
Fragen bei Antragstellung	Bedingungen für einen Rehabilitationsantrag Psychosoziale Faktoren
Entscheidungsfaktoren für und gegen eine Reha	(Potenzielle) Rehabilitand*innen Rehabilitationsbedarf Psychosoziale Faktoren
Ablehnungsgründe	Bedingungen für einen Rehabilitationsantrag

*Anmerkung: *Alle für den Artikel verwendeten Kategorien tauchten in den Kategorienbäumen beider Studien auf. Durch die strukturierende Inhaltsanalyse und die unterschiedliche Schwerpunktsetzung der Studien sind sie unterschiedlich benannt und im Kategorienbaum an unterschiedlichen Stellen zu finden. Inhaltlich sind die Kategorien je Zeile vergleichbar

## Ergebnisse

Die folgenden Ergebnisse orientieren sich am Zugangsprozess zur medizinischen Reha. Dieser umfasst (1.) Erkennung eines Reha-Bedarfs, (2.) Beantragung der medizinischen Reha und (3.) Bedarfsermittlung und -feststellung durch die Leistungsträger. Die Zitate der Prozessbeteiligten wurden für eine bessere Lesbarkeit in dieser Arbeit geglättet.

### Herausforderungen bei der Erkennung eines Rehabilitationsbedarfs

Grundsätzlich liegen sowohl bei MmvB als auch bei MRb ähnliche Herausforderungen vor. So wird die Bedarfserkennung für beide Gruppen als problematisch angesehen: Gesundheitsprofessionelle empfehlen und unterstützen eine medizinische Reha nur, wenn sie einen Nutzen im Sinne einer positiven Prognose für die Teilhabe sehen und ihrem Erachten nach eine ausreichende Rehabilitationsfähigkeit gegeben ist. Für MmvB stellt die Nutzenzuschreibung jedoch einen besonders kritischen Aspekt dar: Hier wird medizinische Reha z. T. als wenig sinnvoll erachtet, insbesondere wenn bereits eine Behinderung oder Pflegebedürftigkeit besteht und keine Erwerbstätigkeit auf dem allgemeinen Arbeitsmarkt angestrebt oder erwartet wird. Dabei spielen auch fehlendes Wissen bzgl. der Potenziale von Reha und Informationsdefizite hinsichtlich des Zugangsprozesses eine Rolle, was u. a. mit einer mangelnden Unterstützung bei der Antragstellung einhergeht. Besonders bei MmvB stellen die Zieldefinition und die Reha-Prognose eine Herausforderung dar, wenn bereits eine Teilhabeeinschränkung vorliegt und eine geringe Selbstständigkeit der Inanspruchnahme einer medizinischen Reha entgegensteht. Gleichzeitig müssen Mediziner*innen für die Erkennung eines Reha-Bedarfs eine Reha-Motivation attestieren, da dies eine entscheidende Voraussetzung für den Zugang zur Maßnahme und deren Erfolg darstellt.

Da An- und Zugehörige sowie hausärztliche Praxen häufig den Anstoß zur Beantragung medizinischer Reha geben, ist ihre Unterstützung zentral für beide Gruppen. Aufgrund ihres höheren Unterstützungsbedarfs im Alltag gilt dies in besonderem Maße für Menschen mit komplexen oder kognitiven Beeinträchtigungen sowie erhöhtem Pflegebedarf. Dies wird durch die folgenden Zitate aus den beiden Studien verdeutlicht:

MeReMBe: *„Das steht und fällt natürlich auch mit der Begleitung des Ganzen. Also, da ist es auch wieder so, wenn das Betreuungsumfeld in der Regel Angehörige sind, … dann ist die Verfolgung der Rehaempfehlung in der Regel besser, als wenn es, … wieder aus dem stationären Wohnbereich ist, wo, … das dann ja über Dritte auf den Weg gebracht werden muss. Also, meistens geht der Brief dann an den Hausarzt, wenn es ein engagierter Hausarzt ist, der dann in den Hausbesuchen das auch weiterverfolgt. (lacht) Dann, ne, dann geht das gut auf den Weg“* (Experteninterview 10, Casemanagerin).

FöBeR: *„Aber dass da auf ärztlicher Seite schon extreme Informationsdefizite sind, wie so ein Antrag überhaupt gestellt wird. Und wenn die diese Informationsdefizite beim Arzt schon da sind, ist es halt sehr unwahrscheinlich, dass er seine Patienten dahingehend unterstützt“* (Online-Fokusgruppe 2, Qualitätsmanagerin in der hausärztlichen Versorgung).

### Herausforderungen bei der Antragstellung zur medizinischen Rehabilitation

Sowohl das Antragsverfahren der GKV als auch das der DRV werden aus Expert*innensicht von MmvB als auch von MRb aufgrund des bürokratischen Aufwands sowie fachspezifischer Begrifflichkeiten als komplex und schwer verständlich wahrgenommen. Dies verursache viele Fehler, z. B. weil die Mediziner*innen die Verschriftlichung der Befunde und das Schreiben des Antrags an Praxispersonal übergeben, welches nicht über den Zweck der Reha informiert oder hierfür nicht geschult sei. MmvB scheinen diesbezüglich zusätzlichen Herausforderungen gegenüberzustehen: Die standardisierten Antragsformulare bilden die komplexen Versorgungsbedarfe oder Mehrfachdiagnosen der MmvB nicht ausreichend ab. Dies habe zur Folge, dass Anträge z. T. unvollständig, ungenau oder fehlerhaft seien. Hinzu kommt, dass Menschen mit kognitiven Beeinträchtigungen möglicherweise aufgrund einer mangelnden Lese- und Schreibkompetenz auf die Hilfe Dritter angewiesen sind. Auch die Kommunikation von Symptomen und Bedarfen kann für kognitiv beeinträchtigte Personen eine Barriere darstellen. Potenziell werden hierfür mehr zeitliche Ressourcen benötigt. Auch eine informierte bzw. partizipative Entscheidungsfindung wird in dieser Gruppe nur eingeschränkt umgesetzt, da ihre Entscheidungen häufig fremdbestimmt getroffen werden und maßgeblich von den Bemühungen ihrer An- und Zugehörigen abhängen.

Weitere Herausforderungen können durch unklare Zuständigkeiten der Leistungsträger entstehen: Bei MmvB wird besonders häufig zunächst eine Zuständigkeit der GKV angenommen, was teils auf der Annahme beruht, sie seien nicht auf dem allgemeinen Arbeitsmarkt tätig. Diese pauschale Zuordnung ist problematisch, da MmvB in vielfältiger Weise auf dem allgemeinen Arbeitsmarkt und nicht ausschließlich in „Werkstätten für behinderte Menschen“ (WfbM) beschäftigt sind.

MeReMBe: *„Ja (I1: okay), da kann ich nur gar nichts zu sagen, weil das Aufgabengebiet bei der Krankenkasse liegt, das heißt also für Menschen mit Behinderung, Menschen mit Beschäftigung in Werkstätten für Behinderte, die Anträge werden weitergeleitet an die Krankenkasse, … Bei uns geht es eben darum, die Versicherten wieder fit zu machen für den allgemeinen Arbeitsmarkt … und wenn wir Beschäftigte, … auf dem zweiten Arbeitsmarkt haben, ist die Zuständigkeit der Rentenversicherung nicht mehr gegeben“* (Experteninterview 3, Referentin eines Leistungsträgers).

FöBeR: *„Weil ich in manchen oder manchmal in meiner Karriere schon mich selbst gefragt habe, warum bekommt der Eine eine Reha sofort zugesprochen und der Andere, wo ich viel mehr Grund sehe und einen viel ausführlicheren und fundierten Reha-Antrag gestellt habe, bekommt sie nicht. …“* (Experteninterview 2, Hausarzt).

### Herausforderungen bei der Bedarfsermittlung

Nach Einreichung des Reha-Antrags erfolgt aufseiten der Leistungsträger die Bedarfsermittlung, an deren Ende die Leistungsentscheidung steht. Diese basiert im Rahmen der medizinischen Reha maßgeblich auf den im Reha-Antrag dargelegten Diagnosen, Befunden und Bedarfen. In diesem Zusammenhang berichteten die Expert*innen in beiden Studien, dass niedergelassene Praxen nur über begrenzte zeitliche, diagnostische und kontextbezogene Ressourcen verfügen, um die Antragstellung dahin gehend angemessen zu unterstützen. Bestehende atypische, multiple oder paradoxe Symptombilder bei MmvB und MRb erschweren zudem eine präzise Diagnosestellung. Es kommt hinzu, dass die genutzten standardisierten, diagnostischen Assessments für Menschen mit schweren kommunikativen oder motorischen Beeinträchtigungen oftmals weder ausreichend validiert noch nutzbar sind. Ein weiteres Problem ist laut Expert*innen das Phänomen des *Diagnostic Overshadowing*, was dazu führen kann, dass vorbestehende Behinderungen in der Diagnosestellung im Vordergrund stehen, während Komorbiditäten oder Sekundärerkrankungen übersehen werden. Dadurch entstehen unvollständige oder unzureichend differenzierte Diagnosen, die die Bedarfsermittlung für Leistungsträger erschweren können.

Die Leistungsentscheidung wird in einem Leistungsbescheid kommuniziert. Aus Expert*innensicht erfolge generell häufig eine Ablehnung des Reha-Antrags, wenn Reha-Ziele, -Prognosen und Therapieoptionen unzureichend beschrieben wurden. Beide Studien zeigen, dass eine fehlende Reha-Fähigkeit oder eine unzureichende Beschreibung der Teilhabeeinschränkungen ebenfalls Gründe für die Ablehnung eines Antrags sein können. Für beide Gruppen wurde berichtet, dass die Ablehnung z. T. wenig transparent, unpräzise bzw. widersprüchlich formuliert werde. Dies sei darauf zurückzuführen, dass Leistungsbescheide auf standardisierten Formulierungen basieren, welche keine individuellen Erläuterungen zulassen. Dabei werden die Entscheidungen von MmvB oft als diskriminierend wahrgenommen, wenn Ablehnungsschreiben auf unvollständigen Sachverhaltsermittlungen beruhen und nicht auf strukturelle Angebotslücken eingehen. Das vermittele den Eindruck, rehabilitative Maßnahmen seien nicht mehr sinnvoll, wenn bereits eine Behinderung vorliegt.

Mit der Leistungsentscheidung geht i. d. R. auch eine Indikations- bzw. Einrichtungszuordnung einher. Dabei erschweren das bestehende, weitgehend indikationsspezifische und nach Erkrankungsgruppen strukturierte Reha-System die Zuordnung. Auch die dem Reha-Bedarf zugehörige gesundheitliche Beeinträchtigung ist bei MmvB nicht klar abgrenzbar von weiteren Erkrankungen. Aus Sicht der Expert*innen ist die medizinische Reha oft nicht ausreichend an den individuellen Bedürfnissen und Zielen der Rehabilitand*innen ausgerichtet und damit insbesondere für Personen mit spezifischen Versorgungsbedarfen zu wenig teilhabeorientiert. MRb haben zudem oftmals nicht ausreichend Kenntnisse über die Kliniken sowie deren Angebote und beziehen Informationen überwiegend aus Internetbewertungen. Teilweise haben sie spezifische Erwartungen an die Kliniken und zeigen sich ggf. enttäuscht, wenn eine Zuweisung zur Wunschklinik nicht möglich ist. Standardisierte Indikationssettings und vielfältige Anforderungen hinsichtlich der Diversität und Lebenssituation (z. B. bei unzureichender Lese- und Schreibkompetenz, Sprachbarrieren oder pflegenden Angehörigen) erschweren die Auswahl der Reha-Einrichtung für MRb und MmvB.

MeReMBe:* „Ja, das ist ja so, so wirklich abhängig, was für eine Behinderung vorliegt … und dann muss man immer noch unterscheiden. … Und dann kommt es eben wirklich darauf an, was ist jetzt der Hauptgrund für/für die Rehabilitation? … Schwieriger wird es dann bei ANDEREN Indikationen, wo auch der pflegerische/also/also personell der//pflegerisch das nicht so groß aufgestellt ist. Weil man davon ausgeht, meinetwegen in der orthopädischen oder kardiologischen Reha, dass die Patienten sich in der Regel selbst versorgen können oder auch im Wesentlichen selbst versorgen können“* (Experteninterview 8, Referentin des Medizinischen Diensts).

FöBeR: *„Leider ist es ja in der Praxis so, dass wir immer über medizinische Aspekte sprechen und es ist eigentlich in vielen Indikationen so, dass die Leute ja auch schon eine Ärztehorde hinter sich haben. In der Orthopädie sind die schon bei fünf, sechs, sieben Ärzten gewesen, in mehreren Kliniken gewesen. So, niemand hat ihnen geholfen. Und dann ist die Reha eigentlich nochmal so ein weiterer Rettungsanker, nochmal ein Versuch. Aber immer unter dem medizinischen Aspekt. Die Frage, ob man trotz dieser orthopädischen Einschränkung nicht trotzdem in der Lebenswelt Verbesserung erfahren kann, nämlich Krankheitsfolgenbewältigung, die werden überhaupt nicht diskutiert mit dem Menschen. Das ist das Problem. …“* (Online-Fokusgruppe 3, Hochschullehrender).

## Diskussion

Im Zugangsprozess zur medizinischen Reha lassen sich für MmvB und MRb sowohl Gemeinsamkeiten als auch Unterschiede in den Herausforderungen feststellen.

Soziale Unterstützung (z. B. durch Gesundheitsprofessionelle, das private Umfeld) erweist sich für den Zugang zur medizinischen Reha für MmvB und MRb zentral. Die Ergebnisse weisen wie auch frühere Studien darauf hin, dass Gesundheitsprofessionelle mehr Wissen über Potenziale medizinischer Reha sowie über das Antragsverfahren benötigen, um Menschen mit Reha-Bedarf beim Antragsprozess angemessen unterstützen zu können [25, 26]. Der bürokratische Ablauf und die Koordination der Antragstellung wurden als herausfordernd, wenig transparent und fehleranfällig beschrieben. Eine mögliche Unterstützung wäre, Beratungsmöglichkeiten durch Lots*innen und Case-Manager*innen zu schaffen, die den Zugang zur medizinischen Reha koordinieren [27]. Ebenfalls bedarf es mehr niedrigschwelliger Informationen, etwa durch Dokumente in leichter Sprache oder offensivere Informationsvermittlung in Praxen [18].

Außerdem besteht die Herausforderung, dass durch die indikationsspezifischen, stationären Reha-Kliniken individuelle Versorgungspfade für beide Gruppen nicht ausreichend umsetzbar sind. Diese gelten jedoch in der Strategie „Reha 2030“ der Weltgesundheitsorganisation (WHO) als essenziell [3] und sollten daher dringend berücksichtigt werden. Gleichzeitig sind diversitätsbezogene und lebenslagenbezogene Bedarfe relevant. Sie können Bedürfnisse, Vorstellungen und Anforderungen an den Zugang zu Rehabilitation beeinflussen [1, 6]. Aus einer intersektionalen Perspektive vergrößern sich die Herausforderungen im Zugang zur medizinischen Reha, wenn mehrere Merkmale (z. B. hohes Alter, weibliches Geschlecht und ein Migrationshintergrund) gleichzeitig vorliegen [6]. Vergleichbare Befunde wurden bereits im Zusammenhang mit der pflegerischen Versorgung von Menschen mit Migrationshintergrund beschrieben [28]. Bei MmvB können neben komplexen Reha-Zielen, Prognoseunsicherheiten und Problemen bei der Antragstellung weitere intersektionale Faktoren den Zugang zur Reha zusätzlich erschweren. Diese Herausforderungen hemmen den Zugang zur medizinischen Reha, der essenziell für eine gleichberechtigte Teilhabe an der Gesundheitsversorgung ist.

Die gesetzlichen Grundlagen der Chancengleichheit für Menschen mit Behinderungen sind in der UN-BRK sowie im Benachteiligungsverbot des § 33c SGB I verankert und umfassen insbesondere das Diskriminierungsverbot und die Gleichheit vor dem Gesetz. Dazu gehören der gleichberechtigte Zugang zu Gesundheits- und Rehabilitationsleistungen, die barrierefrei gestaltet sein müssen, sowie bedarfsgerechte, frühzeitige und wohnortnahe Maßnahmen zur Förderung von Selbstbestimmung, Unabhängigkeit und Teilhabe. Im SGB ist das Ziel der medizinischen Reha bei Menschen mit Behinderungen oder von Behinderung bedrohten Menschen festgelegt. Es dient dazu, Einschränkungen der Erwerbsfähigkeit und Pflegebedürftigkeit zu vermeiden, zu überwinden, zu mindern und eine Verschlimmerung zu verhindern (§ 42 Abs. 1 SGB IX). Damit wird konkret auf den Begriff der Behinderung Bezug genommen, sodass die Relevanz der Versorgung mit rehabilitativen Leistungen auch für MmvB gegeben ist. Diese gesetzlichen Regelungen bilden zwar die Basis für Chancengleichheit, führen aber nicht zwangsläufig zu einer gerechten Versorgung für MmvB sowie zu einer diversitätssensiblen und gerechten Versorgung aller Gruppen, da unterschiedliche Bedarfe und Barrieren nicht berücksichtigt werden [6, 29].

Sowohl MRb als auch MmvB sind aufgrund ihrer Diversitätsmerkmale bzw. Beeinträchtigung und der damit einhergehenden Herausforderungen im Zugangsprozess oftmals auf die Unterstützung ihres sozialen Umfelds angewiesen: Der Zugangsprozess ist ein administratives Verfahren basierend auf komplexen und bürokratischen Richtlinien. Von potenziellen Rehabilitand*innen wird jedoch eine hohe Eigenverantwortung bei der Beantragung einer medizinischen Reha erwartet. Dies macht deutlich, dass das derzeitige Reha-System nicht ausreichend auf individuelle und spezifische Bedarfe ausgerichtet ist. Entsprechend sollte der Zugang zur medizinischen Reha in Zukunft transparenter gestaltet und eine personenzentrierte Unterstützung stärker in die bestehende Gesundheitsversorgung integriert werden [30].

Zukünftige Studien sollten untersuchen, welche Gelingensbedingungen in verschiedenen Gruppen bestehen und wie diese zur Verbesserung des Zugangs zur medizinischen Reha weiterentwickelt werden können. Dabei gilt es, besonders die Förderung individueller Bedarfe in den Blick zu nehmen [31].

### Methodische Reflexion.

Für die Analyse wurden 2 Studien herangezogen, in denen qualitative Interviews und Fokusgruppen mit Expert*innen bzw. am Zugangsprozess zur Reha beteiligten Personen anhand unterschiedlicher Leitfäden durchgeführt wurden. Da in der *FöBeR*-Studie allgemein MRb eingeschlossen wurden, sind darin teilweise auch Informationen zu MmvB enthalten. Der Vergleich der beiden Gruppen wird dadurch jedoch nicht beeinträchtigt, da MmvB nicht im Fokus der Studie standen und ihr Anteil geringer ist.

Obwohl die Studie *MeReMBe* den gesamten Reha-Prozess und die *FöBeR*-Studie die förderlichen Faktoren bei der Beantragung der medizinischen Reha fokussierte, berichteten die Expert*innen beider Studien über erhebliche, indikationsübergreifende Herausforderungen beim Zugang. Eine Stärke der vorliegenden Synthese liegt in der multiprofessionellen Perspektive, die es erlaubt, die Sichtweisen zentraler Versorgungsbeteiligter abzubilden. Hausärztliche Praxen sind für den Zugang zu medizinischer Reha zentral. Allerdings nahmen nur wenige Praxen an Interviews und Fokusgruppen teil, sodass diese essenzielle Perspektive nur unzureichend berücksichtigt werden konnte. Ebenso wurden zusätzliche Unterstützungsangebote, etwa Expert*innen aus der Eingliederungshilfe, im Rahmen der Rekrutierung nicht erreicht.

Pandemiebedingt konnten Fokusgruppen teilweise ausschließlich online durchgeführt werden. Dieser niedrigschwellige Zugang begünstigte die Vielfalt der Prozessbeteiligten bzw. der Expert*innen in der *FöBeR*-Studie. Weitere Publikationen aus der Perspektive der (potenziellen) Rehabilitand*innen befinden sich derzeit in Arbeit.

## Fazit

Ein gerechter Zugang zur medizinischen Reha leistet einen entscheidenden Beitrag zur Verbesserung der gesundheitlichen Versorgung und Teilhabe von MRb. Die für MmvB formulierten Herausforderungen gelten nicht ausschließlich für diese Gruppe, sondern zeigen mögliche Hemmnisse für Rehabilitand*innen mit verschiedenen Diversitätsmerkmalen auf. Von den vorliegenden Erkenntnissen und Implikationen können somit nicht nur MmvB profitieren – diese sind gleichermaßen für die rehabilitative Versorgung aller Menschen mit Reha-Bedarf relevant.

## Data Availability

Für die MeReMBe-Studie und FöBeR-Studie sind die anonymisierten Daten mit einer nachvollziehbar begründeten Anfrage bei den Autor*innen verfügbar.
